# How are socio-demographic and psycho-social factors associated with the prevalence and chronicity of severe pain in 14 different body sites? A cross-sectional population-based survey

**DOI:** 10.1007/s00508-017-1223-x

**Published:** 2017-06-20

**Authors:** Thomas Ernst Dorner, Katharina Viktoria Stein, Julia Hahne, Florian Wepner, Martin Friedrich, Ellenor Mittendorfer-Rutz

**Affiliations:** 1 Institute of Social Medicine, Centre for Public Health, Medical University of Vienna, Kinderspitalgasse 15/I, 1090 Vienna, Austria; 2 International Foundation for Integrated Care, The Quorum, Oxford Business Park North, Garsington Road, 7200 Oxford, UK; 3 Department of Orthopaedic Pain Management, Spine Unit, Centre of Excellence for Orthopaedic Pain Management, Speising, Speisinger Straße 109, 1130 Vienna, Austria; 4 Department of Clinical Neuroscience, Division of Insurance Medicine, Karolinska Institutet, Berzelius väg 3, 17177 Stockholm, Sweden

**Keywords:** Epidemiology, Socio-economic, Chronic pain, Body location, Depression

## Abstract

**Background:**

Severe pain and chronic pain have a high impact on individuals and society. Body location of pain is important with regard to perception, articulation, and underlying biological, mental or social causes of pain.

**Methods:**

A cross-sectional survey was performed in the general Austrian population with 15,474 personally interviewed subjects aged 15 years and older.

**Results:**

The 1‑year period prevalence of severe pain in any body site was 38.6% and of chronic pain 24.9%. In all, 8.1% had pain in at least three body sites. Subjects aged 65 years and older (52.2%), those with low education (43.4%), unemployed subjects (50.4%), retired subjects (52.4%), those with anxiety/depression (67.7%), and subjects with lack of social support (49.6%) were sub-populations with high pain prevalence. In multivariate analyses, depression/anxiety was associated with prevalence and chronicity of severe pain in all body sites (range of ORs 1.89–5.01), while such associations were found for lack of social support (range of ORs 1.33–1.65), female sex (range of ORs 1.38–2.34), higher age (range of ORs 1.09–1.18 for 5 year intervals), as well as low educational (range of ORs 1.47–2.06 primary vs. tertiary education) and unemployment status (range of ORs 1.50–2.62) in most body sites. Being born in non-EU or EFTA states was associated with pain in many body sites (range of ORs 1.38–2.10).

**Conclusions:**

Psychosocial factors are associated with pain presence in similar ways irrespective of location. Regarding socio-demographic factors, differences towards the magnitude and the direction in the association with pain frequency and chronicity in different body sites emerged.

## Background

Pain, especially chronic pain, represents a major public health problem [1, 2] and a frequent reason for health care consultation [3–5] in the adult population. Pain is associated with limited functional capacity [2, 5, 6] and with impaired quality of life [2, 7–9]. Furthermore, pain is associated with high societal costs, both direct and indirect [10, 11]. High levels of loss of productivity [1, 12], sickness absences [13, 14], and disability pension [2, 13, 15, 16] are the main underlying reasons for the indirect costs.

The prevalence of pain is associated with socio-demographic and socio-economic factors. Female sex is commonly reported to be a risk factor for pain [1, 17, 18]; this is especially pronounced regarding headache [19]. Prevalence of pain increases with increasing age [1, 20]. Furthermore, low socio-economic status measured by education, income, and type of profession is significantly associated with the prevalence of pain [1, 20–23]. Pain is also associated with a variety of psycho-social factors like anxiety and depression [24–29], distress, low social support, or low quality of life [28, 30].

Most studies focussing on social factors related to pain either do not differentiate between pain in different body sites [1, 3, 6, 14, 16, 20, 21, 25–27, 31], or focus narrowly on pain in one specific area like back pain [11, 15, 30, 32, 33], low back pain [7, 10], headache [9, 19, 22], on pain due to a specific underlying pathogenesis [4, 8], a specific disease like migraine [28, 29], or osteoarthritis [5].

Still, location of pain matters in different ways. First, it is particularly the spreading and location of pain that patients perceive and therefore report in clinical settings. The underlying biological causes of pain might often be different in different body sites. Biological factors involved in pain perception have been shown to vary considerably according to different body locations [34]. However, the perception of pain and the development of chronic pain do not depend on biological factors only; here psycho-social and socio-economic factors contribute to a notable extent [30, 35, 36]. Pain, and especially chronic pain, is therefore often not only regarded as a symptom of an underlying disease, but also as a disease entity per se [37]. Nevertheless, to date there is limited scientific knowledge on whether the strength of the associations of socio-demographic and psycho-social factors with pain varies across different body locations.

In chronic widespread pain, the importance of physical, psychological, and social factors is well examined [38]. It is defined as chronic pain, affecting the left and right side of the body, and sites above and below the diaphragm, plus pain in the axial skeleton [39]. Hence, it affects at least three different body sites. For the diagnosis of fibromyalgia, one of the main causes of chronic widespread pain, pain must be present in at least three out of 19 body sites [40]. There is however scarce information on how socio-demographic and psycho-social factors are related to widespread pain compared with localised pain.

Knowledge on any differences in associations of these factors with prevalence and chronicity of pain could contribute to tailor-made and person-centred treatment and rehabilitation efforts.

This study aimed to assess the 1‑year prevalence and chronicity of severe pain in the Austrian general population in different body sites, and to analyse associations of socio-demographic and psycho-social factors with pain presence in different body sites and in patients with pain in three or more body sites compared to one or two body sites.

## Methods

The database for the analysis was the Austrian Health Interview Survey (AT-HIS) 2006-07 [41], a repeatedly performed representative survey carried out by Statistics Austria. Its aim is to gain knowledge about subjective health, quality of life, health behaviour, and utilisation of the health care system. The subjects were personally interviewed between March 2006 and March 2007 by trained interviewers. The interviews were conducted face-to-face using CAPI (computer assisted personal interviewing) by a total of 137 interviewers who were comprehensively trained before the start of the survey by personnel at Statistics Austria. The sample was stratified by 32 geographic regions, with the same number of subjects being included from each region (sample size of 770 subjects per region and 933 subjects for the region within the capital Vienna). To balance possible distortions through the geographic stratification of the sample, the data were weighted using the number of people living in each region, age in 5‑year groups, and sex as weight factors. The gross sample size comprised 25,130 people, aged 15 years and older. A total of 9656 subjects were excluded for varying reasons: 5709 subjects refused or discontinued the interview, 3308 were excluded due to difficulties in contacting them or because of deficiency regarding their command of the German language, and for 639 cases data quality was insufficient. The data for a total of 15,474 subjects were eligible for analysis, representing a response rate of 63.1%. Response rates were comparable across regions and age groups, ranging from 52% to 69% [41]. Missing values were systematised, imputations of item-non-response were based on established imputation guidelines based on a fundamental analysis of the non-responses [41]. The questionnaire underlying the interviews was designed based on the European Health Interview Survey (E-HIS) [42] and was adapted for Austria by an expert panel. In addition to the international mandatory questions based on the E‑HIS, national questions were included in the AT-HIS, which also comprised the questions about pain.

**Fig. 1:**
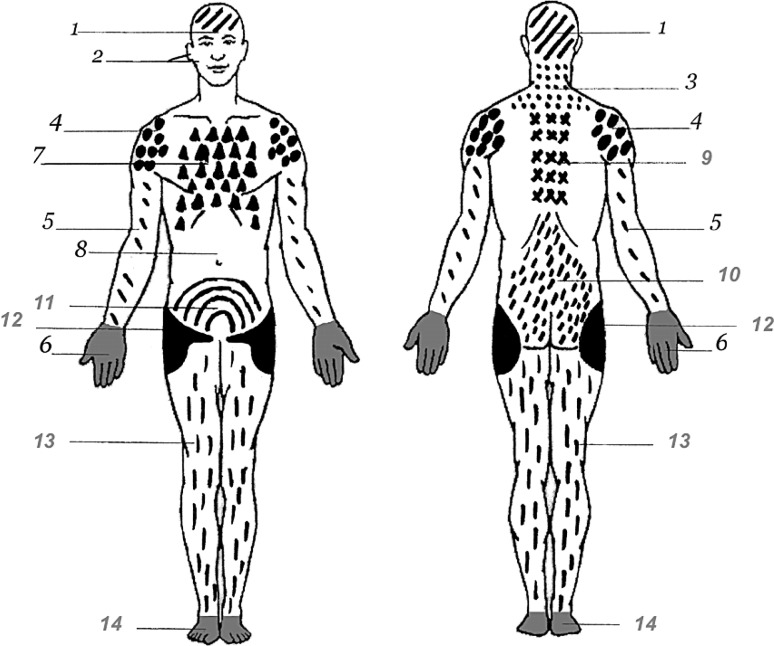
Body image with possible body sites of pain. *1* head, *2* face, *3* neck, *4* shoulder, *5* arm, *6* hand, *7* chest, *8* abdomen, *9* upper back, *10* lower back, *11* pelvis, *12* hip, *13* leg, *14* foot

Regarding pain, the following questions were included in the AT-HIS: “Did you suffer from severe pain in one or more than one body site during the last 12 months?” If this was answered by “yes”, subjects were asked to identify the body site or body sites in which they experienced severe pain on an image of the body. In this image, the front and the back of a body map was shown with a total of 14 body sites (Fig. 1). For each body site with severe pain, subjects were asked, whether the pain had also occurred in this region within the last 7 days. If this was answered by “yes”, the subjects were also asked whether the pain lasted for longer than for 3 months. Chronic pain was considered when the severe pain in the respective body site had occurred in the last 7 days and had lasted for longer than 3 months. This definition is in line with the definition of chronic pain by the International Association for the Study of Pain [43].

Sex, age, country of birth, level of education, and employment status were assessed as socio-demographic variables. Age was recorded in steps of 5 years and categorised into three groups, 15–39 years, 40–64 years, and 65 years and older. Country of birth was measured as born in Austria, in the EU-15 (the member states of the European Union up to 2004, except Austria), countries of the European Free Trade Association (EFTA), EU-27 (the new member states of the European Union from 2004), countries belonging to the Former Yugoslavia (except Slovenia), or any other countries. For the logistic regression analyses, information on country of birth was used with three categories (Austria; states of the European Union [EU] + European Free Trade Association (EFTA); other countries). Level of education was categorised into three levels: primary education (up to the age of 15 years), secondary education (apprenticeship, vocational school or secondary school with the Austrian school leaving exam “Matura”), and tertiary education (university or any other vocational training after the “Matura”). Employment status was assessed in four categories: gainfully employed, unemployed, retired (which also included subjects in early retirement and disability pension), and other (which included subjects in formal education, housewives and househusbands, subjects on maternity or paternity leave, and persons in military service). Anxiety/depression was assessed with the question “Have you ever suffered from anxiety or depression?” with the possible values “yes” and “no”. For measuring social support, the question of the World Health Organization quality of life questionnaire [44] “How satisfied are you with the support you get from your friends?” was used. The possible answer to this ranged on a five-point Likert scale from “very satisfied” to “very dissatisfied” and was dichotomised according to the median which was 2. Multicollinearity was judged if Spearman correlation coefficients were > 0.5.

For statistical analyses, SPSS 20 was used. Bivariate analyses were undertaken by means of cross-tabs, and group differences were assessed with the Pearson’s Chi^2^-test including the z‑test. Moreover, mutually adjusted binary logistic regression models were applied comparing individuals with pain at a specific body site with those without any reported pain at this body site. Having suffered from pain in the respective body sites or chronicity of pain were used as dependent variables and sex, country of birth, educational level, employment status, anxiety/depression, and social support as categorical and age as continuous independent variables. Additionally, a multivariate binary regression model was applied only in those who were suffering from pain in any body site and the dependent variable was having suffered from pain in at least three body sites (vs. only one or two body sites) with the same independent variables as mentioned before. The cut-off of three body sites was chosen because this is the prerequisite for meeting the criteria for chronic widespread pain. The results of the logistic regression models are presented as odds ratios (OR) with 95% confidence intervals (CI).

The secondary analysis of the AT-HIS database that was used for this study was approved by the Ethics Committee of the Medical University Vienna (EC # 770/2011).

## Results

The 1‑year period prevalence of severe pain in any body site was 38.6% in the whole sample (35.4% in men and 41.5% in women). Among those with severe pain, 64.4% reported chronic pain. Thus, 24.9% were suffering from chronic pain in at least one body site (21.1% in men and 28.4% in women). The most common body sites affected by severe pain were the lower back, followed by the leg, the neck, and the head. The estimates for 1‑year prevalence of severe pain in the respective body sites are shown in Table 1 for men and women. Pain occurred significantly more often in women than in men in almost all body sites (Table 1).

**Table 1:** 1-year period prevalence of severe pain and proportion of chronicity in those with severe pain in the last 12 months in different body sites in 7452 men and 8021 women aged 15 years and older in the general Austrian population

	1-year period prevalence (%)			Chronicity^a^ (%)		
	Total	Men	Women	Total	Men	Women
Head	6.6	4.1	9.0**	56.9	53.2	58.5
Face	1.6	1.3	1.9*	37.1	35.4	38.3
Neck	7.2	5.4	9.0**	66.7	63.4	68.5
Shoulder	6.1	5.7	6.5	70.0	67.0	72.3
Arm	3.1	2.5	3.7**	69.8	62.4	74.5*
Hand	3.8	2.5	5.1**	76.1	67.0	80.1**
Chest	1.8	1.9	1.7	58.4	57.9	58.6
Abdomen	2.8	2.2	3.3**	51.9	44.6	56.4*
Upper back	5.4	4.7	6.1**	69.4	64.9	72.6*
Lower back	15.6	15.0	16.1*	65.7	60.7	70.0**
Pelvis	1.7	1.2	2.2**	39.8	43.7	37.7
Hip	4.2	3.3	5.1**	73.9	72.8	74.7
Leg	9.0	8.6	9.3	69.7	64.5	74.1**
Foot	4.7	4.1	5.2*	66.5	60.1	71.3*
**Any body site**	**38.6**	**35.4**	41.5**	**64.4**	**59.5**	68.3**
**At least 3 body sites**	**8.1**	**6.5**	9.6**	**93.2**	**93.2**	**93.1**

^a^Chronicity was defined as severe pain in the respective body site that had occurred in the last 7 days and had lasted for longer than 3 months

*P < 0.05; **P < 0.001

There were significant differences (*P* < 0.001) found in the 1‑year period prevalence in different subgroups. Prevalence increased with age and was 26.6%, 43.6%, and 52.2% in the three age groups, respectively. People born in Turkey and in eastern EU states had the highest prevalence (45.9% and 45.0%, respectively). The prevalence decreased with increasing level of education and was 43.4%, 37.7%, and 30.5% in the three levels of education, respectively. Retired (53.4%) and unemployed (50.4%) subjects had a significantly higher 1‑year period prevalence of severe pain than gainfully employed subjects (33.7%). Those with anxiety/depression and those with lack of social support also had a higher prevalence (67.7% vs. 36.4%, and 49.6% vs. 36.6%, respectively). With regard to the different body sites, the associations of socio-demographic factors with the prevalence of pain, based on multivariate models, varied to a considerable degree (Tables 2, 3, and 4).

**Table 2:** Multivariate odds ratios (ORs)^a^ and 95% Confidence interval (CI) of socio-demographic and psycho-social factors associated with 1‑year period prevalence of severe pain in different body sites of the spine in the Austrian population aged 15 years and older

	Neck		Upper back		Lower back	
	OR	95% CI	OR	95% CI	OR	95% CI
*Sex*						
Men	1	–	1	–	1	–
Women	1.73	1.51–1.98	1.26	1.09–1.46	1.09	0.99–1.19
Age (5 year intervals)	1.09	1.06–1.12	1.10	1.07–1.13	1.12	1.10–1.15
*Country of birth*						
Austria	1	–	1	–	1	–
EU+EFTA^b^	0.81	0.61–1.06	1.05	0.78–1.41	0.96	0.79–1.17
Other	1.22	0.99–1.50	1.48	1.18–1.85	0.96	0.82–1.12
*Education*						
Primary	0.85	0.67–1.09	1.57	1.15–2.15	1.47	1.21–1.79
Secondary	0.96	0.77–1.20	1.43	1.07–1.91	1.60	1.34–1.91
Tertiary	1	–	1	–	1	–
*Employment*						
Gainfully employed	1	–	1	–	1	–
Unemployed	1.47	1.08–1.99	0.97	0.66–1.41	1.51	1.21–1.89
Retired	0.96	0.78–1.18	0.89	0.71–1.13	0.79	0.68–0.91
Other	0.74	0.61–0.90	0.57	0.44–0.73	0.60	0.51–0.70
*Anxiety/depression*						
No	1	–	1	–	1	–
Yes	2.61	2.19–3.11	3.00	2.48–3.63	2.18	1.89–2.52
*Lack of social support*						
No	1	–	1	–	1	–
Yes	1.33	1.14–1.55	1.42	1.20–1.68	1.44	1.29–1.61

^a^Results of mutually adjusted multivariate binary regression models

^b^Member states of the European Union until 2007 plus European Free Trade Association: Belgium, Bulgaria, Cyprus, Czech Republic, Denmark, Estonia, Finland, France, Germany, Greece, Hungary, Ireland, Island, Italy, Latvia, Liechtenstein, Lithuania, Luxembourg, Malta, The Netherlands, Norway, Poland, Portugal, Romania, Slovakia, Slovenia, Spain, Sweden, Switzerland, United Kingdom

**Table 3:** Multivariate odds ratios (ORs)^a^ and 95% Confidence interval (CI) of socio-demographic and psycho-social factors associated with 1‑year period prevalence of severe pain in different body sites of the upper and lower extremities in the Austrian population aged 15 years and older

	Shoulder		Arm		Hand		Hip		Leg		Foot	
	OR	95% CI	OR	95% CI	OR	95% CI	OR	95% CI	OR	95% CI	OR	95% CI
*Sex*												
Men	1	–	1	–	1	–	1	–	1	–	1	–
Women	1.06	0.92–1.22	1.21	0.99–1.45	1.82	1.51–2.19	1.38	1.16–1.64	0.90	0.79–1.01	1.08	0.91–1.26
Age (5 year intervals)	1.17	1.14–1.21	1.19	1.14–1.23	1.16	1.12–1.20	1.18	1.14–1.23	1.18	1.15–1.21	1.14	1.11–1.18
*Country of birth*												
Austria	1	–	1	–	1	–	1	–	1	–	1	–
EU+EFTA^b^	1.11	0.85–1.46	0.52	0.32–0.85	1.21	0.87–1.67	0.95	0.68–1.33	0.83	0.64–1.07	0.88	0.62–1.23
Other	1.67	1.36–2.07	1.48	1.11–1.98	1.09	0.81–1.47	1.61	1.23–2.11	1.38	1.14–1.66	1.48	1.16–1.88
*Education*												
Primary	1.07	0.81–1.41	1.64	1.05–2.56	1.73	1.17–2.57	1.64	1.10–2.46	1.70	1.30–2.22	2.06	1.41–3.02
Secondary	1.20	0.93–1.55	1.55	1.02–2.38	1.61	1.10–2.34	1.72	1.17–2.54	1.58	1.23–2.03	1.81	1.26–2.61
Tertiary	1	–	1	–	1	–	1	–	1	–	1	–
*Employment*												
Gainfully employed	1	–	1	–	1	–	1	–	1	–	1	–
Unemployed	1.22	0.87–1.72	2.21	1.47–3.33	0.98	0.60–1.60	2.12	1.41–3.19	1.87	1.40–2.48	1.63	1.12–2.38
Retired	0.73	0.58–0.90	0.86	0.64–1.17	0.83	0.63–1.09	1.65	1.26–2.16	1.22	1.01–1.47	1.18	0.92–1.51
Other	0.78	0.62–0.97	1.20	0.89–1.61	0.85	0.65–1.11	1.07	0.80–1.44	1.21	1.00–1.46	1.03	0.80–1.33
*Anxiety/depression*												
No	1	–	1	–	1	–	1	–	1	–	1	–
Yes	2.63	2.18–3.17	3.55	2.83–4.45	3.31	2.68–4.09	1.89	1.52–2.36	2.46	2.09–2.90	2.75	2.25–3.37
*Lack of social support*												
No	1	–	1	–	1	–	1	–	1	–	1	–
Yes	1.09	0.92–1.30	1.53	1.24–1.89	1.23	1.00–1.51	1.63	1.36–1.96	1.15	1.00–1.33	1.20	1.00–1.45

^a^Results of mutually adjusted multivariate binary regression models

^b^Member states of the European Union until 2007 plus European Free Trade Association: Belgium, Bulgaria, Cyprus, Czech Republic, Denmark, Estonia, Finland, France, Germany, Greece, Hungary, Ireland, Island, Italy, Latvia, Liechtenstein, Lithuania, Luxembourg, Malta, The Netherlands, Norway, Poland, Portugal, Romania, Slovakia, Slovenia, Spain, Sweden, Switzerland, United Kingdom

**Table 4:** Multivariate odds ratios (ORs)^a^ and 95% Confidence interval (CI) of socio-demographic and psycho-social factors associated with 1‑year period prevalence of severe pain in different body sites of trunk in the Austrian population aged 15 years and older

	Head		Face		Chest		Abdomen		Pelvis	
	OR	95% CI	OR	95% CI	OR	95% CI	OR	95% CI	OR	95% CI
*Sex*										
Men	1	–	1	–	1	–	1	–	1	–
Women	2.34	2.03–2.70	1.41	1.08–1.83	0.78	0.61–1.01	1.46	1.19–1.80	1.92	1.47–2.50
Age (5 year intervals)	0.95	0.92–0.97	0.97	0.93–1.02	1.14	1.08–1.20	1.01	0.97–1.05	0.93	0.88–0.98
*Country of birth*										
Austria	1	–	1	–	1	–	1	–	1	–
EU+EFTA^b^	0.63	0.46–0.87	0.77	0.43–1.36	0.55	0.29–1.04	0.88	0.57–1.35	0.74	0.42–1.31
Other	1.09	0.89–1.34	0.94	0.62–1.42	2.10	1.49–2.96	1.48	1.12–1.97	0.96	0.63–1.48
*Education*										
Primary	0.91	0.71–1.17	0.64	0.41–1.02	1.71	0.93–3.15	0.88	0.60–1.28	0.66	0.39–1.10
Secondary	0.86	0.69–1.08	0.70	0.47–1.04	2.03	1.13–3.63	0.93	0.66–1.32	1.15	0.73–1.81
Tertiary	1	–	1	–	1	–	1	–	1	–
*Employment*										
Gainfully employed	1	–	1	–	1	–	1	–	1	–
Unemployed	2.00	1.49–2.59	2.25	1.38–3.67	0.94	0.51–1.75	2.56	1.77–3.71	1.04	0.52–2.08
Retired	0.95	0.75–1.19	1.00	0.64–1.54	1.00	0.68–1.47	1.09	0.79–1.52	2.07	1.36–3.16
Other	0.92	0.77–1.10	0.96	0.67–1.38	0.89	0.59–1.35	1.03	0.77–1.37	1.12	0.79–1.59
*Anxiety/depression*										
No	1	–	1	–	1	–	1	–	1	–
Yes	3.73	3.11–4.47	3.58	2.57–4.97	5.01	3.81–6.59	4.24	3.38–5.40	3.69	2.69–5.07
*Lack of social support*										
No	1	–	1	–	1	–	1	–	1	–
Yes	1.41	1.20–1.66	1.59	1.17–2.15	1.65	1.26–2.16	1.34	1.06–1.70	1.49	1.11–2.01

^a^Results of mutually adjusted multivariate binary regression models

^b^Member states of the European Union until 2007 plus European Free Trade Association: Belgium, Bulgaria, Cyprus, Czech Republic, Denmark, Estonia, Finland, France, Germany, Greece, Hungary, Ireland, Island, Italy, Latvia, Liechtenstein, Lithuania, Luxembourg, Malta, The Netherlands, Norway, Poland, Portugal, Romania, Slovakia, Slovenia, Spain, Sweden, Switzerland, United Kingdom

In subjects with severe pain in at least one body site, the following parameters were significantly associated with suffering from severe pain in at least three body sites (vs. in only one or two): female sex, OR: 1.26 (95% CI: 1.10–1.44); higher age, OR per 5 years: 1.06 (1.04–1.09); being born in countries other than the EU or EFTA vs. Austria, OR: 1.57 (1.27–1.94); unemployed, OR: 1.59 (1.17–2.15); and retired people, OR: 1.46 (1.18–1.80) vs. gainfully employed; anxiety/depression, OR: 2.95 (2.49–3.50); and lack of social support, OR: 1.28 (1.10–1.49).

All body sites showed a high proportion of chronicity, i. e that severe pain had occurred in the last 3 months (including the last week). Chronicity of pain varied in the respective body sites between 37.1% in the face and 76.1% in the hand. A higher proportion of chronicity for women could be found regarding pain in the extremities, in the back, and in the abdomen. In those with severe pain in at least three body sites, almost all were suffering from chronic pain (Table 1). Moreover, multivariate regression analyses revealed female sex to be associated with a higher risk for chronicity regarding pain in the hand and abdomen (range of ORs 1.66–1.75), but a lower risk for chronicity regarding headache (OR 0.66). Also associated with higher risk of chronicity in almost all body sites were the factors higher age (range of ORs 1.12–1.27 for 5 year intervals), unemployment (range of ORs 1.18–9.54), depression/anxiety (range of ORs 1.56–3.18), and lack of social support (range of ORs 1.29–1.97). Individuals not born in Austria had a higher risk for chronicity regarding pain in the chest (OR 2.19) and the upper back (OR 2.24). Lowest vs. highest education was associated with a higher risk for chronicity regarding pain in the head, spine, hip and leg (range of ORs 1.99–10.80) (data not shown).

## Discussion

In this study we found high levels of prevalence and of chronicity of severe pain in the Austrian population aged 15 years and older. We found variations in the associations between socio-demographic characteristics with pain frequency and chronicity with respect to the affected body sites. Psycho-social factors like anxiety/depression and lack of social support were strongly associated with pain parameters in all body-sites analysed.

The 1‑year period prevalence of severe pain in our study (35.4% in men and 41.5% in women) appeared to be lower when compared to other studies. Gerdle et al., for example, found a 1-month period prevalence of pain in 57.2% of men and 68.2% of women [1], and Frießem found a point prevalence of pain in primary care of 62.1% [3]. The most likely reasons for these differences in pain prevalence estimates include differences in measures of pain intensity. In the present study, severe pain was assessed, thus pain of low intensity might not have been reported. Proportions of chronicity in our study varied between 60% and 70%, which is relatively high. In primary care, only 40% of patients with pain were found to suffer from chronic pain [3]. Again, the explicit question for “severe” pain might be the reason for this high percentage of chronicity. The proportion of people with chronic pain (21.1% in men and 28.4% in women) is comparable to a European study, where prevalence of chronic pain was found to be 19% in the adult population, and 21% in Austria [2]. In line with this study, we also found the most common painful body locations to be the back, especially the lower back, joints, and the head [2]. The proportion of people suffering from pain in at least three regions (6.5% of men and 9.6% of women) is comparable to the prevalence of widespread pain in other studies (e. g. around 10% in the UK) [45].

Female sex was associated with a higher risk of being affected by severe pain in most body sites. This was especially pronounced regarding headache, pain in the pelvis, and neck pain. Additionally, female sex was associated with a higher risk for chronicity, especially regarding abdominal pain and pain in the hand. A higher prevalence of pain in women is in line with other studies regarding pain epidemiology [1, 19, 46]. However, in our study, men were more often affected by chest pain. Differences in the prevalence of the diseases underlying the pain may explain these sex-specific differences. Women are reported to suffer more often from chronic tension headache, migraine, cervicogenic headache, and neck pain [17]. This might explain the higher proportion of women affected by headache and neck pain. The higher risk of suffering from pelvic pain in women might be explained by many possible gynaecological reasons for pelvic pain. However, there was also a remarkably high proportion of men with severe pelvic pain in our study. The fact that women seem to more frequently have complaints like carpal tunnel syndrome, as well as chronic constipation and irritable bowel syndrome [17], might contribute to the higher risk of chronicity in women with pain in the hand and abdominal pain, respectively. On the other hand, gout, intermittent claudication, and duodenal ulcers [17] seem to be more frequent in men, as well as classic symptoms of pectoral angina [47]. This might explain the higher risk in men for pain in the legs and chest pain. Still, not only biological, but also psycho-social and cultural aspects have been discussed as contributing to the gender specific differences in pain epidemiology, including role socialisation, cognitive and affective factors, and factors related to coping mechanisms [18].

Higher age was associated with a higher risk for prevalence and chronicity of severe pain in almost all body sites, which is in line with most other studies [1, 46]. However, we found an inverse association between age and pelvic pain, and age and headache. Headache has previously been shown to occur equally often in all age groups, including children and adolescents [19]. Reasons for a higher risk for pelvic pain in younger populations might include problems related to sexuality and reproductivity.

Different measures of socio-economic status including low educational level, unemployment, and retirement were related to severe pain in most body sites with some differences in the importance of the various body sites. Low education has been linked to higher pain frequency in earlier studies [20, 48, 49]. Different explanations for this association have been proposed, including higher work stress and physical work load, lower control at work, imbalance between effort and reward [50, 51], or mediation through psycho-social factors and poor mental health status [48, 49, 52]. With regard to the association of retirement and pain frequency, it should be noted that multivariate analyses were controlled for age. It can therefore be assumed that not only old age, but also early retirement and disability are involved in these associations. Additionally, unemployment was associated with pain in almost all body sites. In both cases, retirement and unemployment, pain, especially chronic pain, might have contributed to an exit from the labour market. On the basis of our data, no conclusions can be drawn on whether chronic pain was the cause or the consequence of unemployment or retirement. Still, the longer people are off work due to pain, the lower the likelihood seems to be of their returning to work [53, 54].

In our study, a history of anxiety/depression was strongly associated with pain frequency and chronicity, irrespective of body site. Pain and common mental disorders are strongly interlinked. An association between pain and depression [2], or anxiety has also been found in other studies [24, 25]. In accordance with our study, an association of multiple pain, pain severity, and depressive disorders was found in the elderly population in a recent study in Germany [55]. Moreover, studies suggest that the interaction between pain and mental disorders act synergistically with regard to higher health care utilisation [33], or exit from the labour market [16, 32]. However, whether depression and anxiety are a cause or consequence of pain, or whether they are the results of different paths of the same pathogenesis, still remains unresolved and under discussion [30, 31]. Depression has been shown to predict the onset of pain, and pain has been shown to predict the onset of depression [25, 26]. However, the association between chronic pain and common mental disorders is multifactorial in its nature, including shared neurobiological, psychological, and genetic factors [24, 27, 56].

Besides the association with common mental disorders, a lack of social support was related to pain parameters in almost all body sites. This association underlines the social component in the frame of the bio-psycho-social concept of pain and chronic pain [35]. Social factors like low social trust and low social capital [57] have been shown to be associated with pain, but also with depression and psycho-somatic symptoms [58] and can thus contribute to the enhancement and chronification of pain. Additionally, psycho-social discomfort is reported to be associated with many physical symptoms, including pain in various body sites [59]. Indeed, social support has been shown to help in coping with chronic pain [60, 61], and a lack of social support favours the development of pain [62].

A strength of our study is its design as a nationwide personal interview survey in a central European country. Compared to other studies, two different measurers of pain, 1‑year period prevalence and chronicity, could be analysed in relation to socio-economic and psycho-social parameters in 14 different body sites and compared with each other. The response rate of 61.3% should be mentioned as a possible limitation of our study. Another limitation of the study design relates to its cross-sectional nature. Therefore, any conclusions related to temporal associations cannot be drawn. We conceptualised chronic widespread pain as having pain in at least three body sites, being aware of the fact that this concept does not fully cover the definition of chronic widespread pain.

## Conclusions

In conclusion, we found differences in the association between socio-economic factors with pain frequency and chronicity in different body sites. Female sex and high age were both positively and negatively associated with severe pain in different body sites. There are also remarkably different magnitudes in those associations. The psycho-social factors, i. e. anxiety/depression and lack of social support, were strongly associated with pain presence, irrespective of the location of pain. Additionally, we found that several socio-demographic and psychosocial factors are more strongly associated with patients suffering from pain in three or more body sites compared to patients with pain in only one or two body sites.
